# Editorial: Neuromuscular control modelling of static and dynamic balance maintenance

**DOI:** 10.3389/fneur.2022.1079410

**Published:** 2022-11-22

**Authors:** Alessandro Mengarelli, Federica Verdini, Sandro Fioretti, Recep Ali Ozdemir, Ramona Ritzmann

**Affiliations:** ^1^Department of Information Engineering, Università Politecnica delle Marche, Ancona, Italy; ^2^Department of Neurology, Harvard Medical School, Boston, MA, United States; ^3^Department of Sport Science, University of Freiburg, Freiburg, Germany

**Keywords:** balance maintenance, biosystem modeling, perturbed upright stance, neuromuscular control, post-urography

The study of the upright stance is a rooted research field (1) but it remains fundamental for unveiling how the nervous system tunes and adapts its response to deal with the need for maintaining balance and avoiding falls. The instrumental investigation of human posture is still central for describing the inner characteristics of balance control and the dynamics that drive the optimization and deterioration of postural regulation (1, 2). Indeed, balance impairment is one of the most common symptoms affecting patients suffering from a wide spectrum of disorders, ranging from musculoskeletal to neurological (1). In addition, also elderly people can suffer from upright stance deterioration, which can negatively impact their quality of life, leading to an enhanced risk of falls (1). Although static posture can appear a simple task, it is well-acknowledged that it encompasses both passive and active control dynamics (2), representing perhaps the most common condition under which balance maintenance is investigated (1). On the other hand, the manipulation of the environmental and internal conditions represents one of the most commonly used ways for introducing perturbations within the regulatory dynamics of balance, to elicit and analyze different control strategies with respect to the proper functions of maintaining a static upright stance.

In the posturographic field, several approaches have been proposed and developed throughout the years for investigating the balance control process, ranging from analysis of the kinematics and kinetics quantities that can be recorded during a standing trial, either static or perturbed, to the modeling of the mechanical and regulatory processes that constitute the basis of balance maintenance (2). Far from being mutually exclusive, these approaches can be viewed as complementary ways of enhancing understanding of how the central and peripheral nervous systems manage sensory-motor integration. Findings in this field can be beneficial also clinical and diagnostic viewpoints since complex mechanisms and dynamics are unveiled by using static or dynamic posturography and are likely to be degraded by diseases affecting musculoskeletal and nervous systems.

This Research Topic includes four articles, two of which investigate perturbed balance maintenance by using data-driven modeling approaches (Tigrini et al.; Cherif et al.), one dealt with the agreement between geometric and spectral metrics computed from the center of pressure trajectory (Sozzi, Ghai et al.), and one investigated the vertical component of the ground reaction force (Sozzi, Do et al.).

Tigrini et al. propose to model postural responses to unexpected perturbation of upright stance, provided by the displacement of the base of support along the anterior-posterior direction, through a piecewise autoregressive model that encompasses external input. Hybrid models allowed them to deal with the non-linearity arising when balance undergoes sudden and unexpected perturbations. In passing, switching control policies were advocated for interpreting the regulatory mechanisms underlying postural dynamics (2). In this study the very initial phase of the counterbalancing response was considered for investigating the control dynamics arising in a transient phase, avoiding the long-term postural response, which encompasses voluntary actions. A hybrid control policy, together with a simple upright stance biomechanical model, such as a single-link inverted pendulum, provided a reliable model of the neural regulatory strategies applied to counteract a sudden and unexpected perturbation of balance. In particular, the hybrid control model suggested that different strategies are adopted for different sensory configurations, i.e., when all the sensory information is available, when the visual input is missing, and when a cognitively demanding task is performed.

The study by Cherif et al. investigated balance maintenance from a data-driven modeling approach. In this study, attention was devoted to the role of the voluntary and reflex regulatory activity carried out for controlling standing balance. This work also explores upright stance maintenance, which underwent a series of perturbations provided by a dedicated apparatus and several inter-stimulus time intervals were investigated. To fit the distribution-response data, three models were compared, encompassing both linear and non-linear processes. Outcomes showed that postural response is highly non-linear for short inter-stimulus intervals, and the most suitable model was one characterized by two processes with fixed and variable delays. Such a model is coherent with the idea that two pathways are available for providing input to the motor control system, i.e., a short input involving trans-cortical pathways and a longer input, that can encompass a variable amount of time before being processed into a regulatory stimulus to the motor system.

On the other hand, Sozzi, Ghai et al. focused on the agreement between different domain measures derived from the center of pressure (COP) displacement. Standing trials were performed on both a rigid and a foam surface, and the adaptation of postural response to repeated trials was taken into account. The results of this study contribute to an understanding of which markers of postural stability can be considered reliable tools for clinical balance evaluations. It is worth highlighting that a set of classical COP metrics were considered, thus providing information on highly interpretable quantities, commonly used in clinical evaluations.

Balance maintenance performed on compliant and solid surfaces was also considered in the last work of this collection (Sozzi, Do et al.). The vertical component of the ground reaction force (VGRF) was analyzed, in terms of its amplitude oscillations and spectral characteristics, and a direct comparison with the COP trajectory was undertaken. The results showed the presence of a postural rhythm, based on the spectral properties of the VGRF, which reflects, in turn, a typical muscle activation pattern in proper standing balance either on hard or compliant surfaces. It is noteworthy that this specific characteristic of balance maintenance seems not to be mirrored in COP displacement, and thus likely represents an additional feature that should be considered in human upright stance analysis.

This Research Topic brings together articles on some of the latest advancements in the field of instrumented posturography. It is noteworthy that in all the works standing balance underwent perturbations, highlighting the added value of injecting external disturbances to elicit specific responses from the musculoskeletal and neural control systems. However, the large spectrum of possible perturbations that can be applied to human balance prevents rough generalization of the results, highlighting the need for working toward the definition of a unified perspective in the experimental practice when dealing with upright stance perturbation. Data-driven modeling approaches, adopted in two papers (Tigrini et al.; Cherif et al.), provide evidence regarding the neural pathways involved in postural control over short and long temporal epochs. However, investigating dynamic quantities directly measurable from a standing task still represents a valuable source of information (Sozzi, Ghai et al.; Sozzi, Do et al.), indicating that these two approaches should be considered equally reliable tools for gaining further insights into the motor and neural regulation of balance. Their integration in a unified view can be seen as one of the challenges in the field of human neuro-muscular control.

## Author contributions

All authors listed have made a substantial, direct, and intellectual contribution to the work and approved it for publication.

## Conflict of interest

The authors declare that the research was conducted in the absence of any commercial or financial relationships that could be construed as a potential conflict of interest.

## Publisher's note

All claims expressed in this article are solely those of the authors and do not necessarily represent those of their affiliated organizations, or those of the publisher, the editors and the reviewers. Any product that may be evaluated in this article, or claim that may be made by its manufacturer, is not guaranteed or endorsed by the publisher.
